# Advances in the Research on Plant WRKY Transcription Factors Responsive to External Stresses

**DOI:** 10.3390/cimb45040187

**Published:** 2023-04-01

**Authors:** Hongli Wang, Xi Cheng, Dongmei Yin, Dongliang Chen, Chang Luo, Hua Liu, Conglin Huang

**Affiliations:** 1 College of Ecology, Shanghai Institute of Technology, Shanghai 201418, Chinayindm@sit.edu.cn (D.Y.); 2 Beijing Engineering Research Center of Functional Floriculture, Institute of Grassland, Flowers and Ecology, Beijing Academy of Agriculture and Forestry Sciences, Beijing 100097, China

**Keywords:** WRKY transcription factor, structural characteristics and classification, biological function, plant secondary metabolism, control network

## Abstract

The WRKY transcription factors are a class of transcriptional regulators that are ubiquitous in plants, wherein they play key roles in various physiological activities, including responses to stress. Specifically, WRKY transcription factors mediate plant responses to biotic and abiotic stresses through the binding of their conserved domain to the W-box element of the target gene promoter and the subsequent activation or inhibition of transcription (self-regulation or cross-regulation). In this review, the progress in the research on the regulatory effects of WRKY transcription factors on plant responses to external stresses is summarized, with a particular focus on the structural characteristics, classifications, biological functions, effects on plant secondary metabolism, regulatory networks, and other aspects of WRKY transcription factors. Future research and prospects in this field are also proposed.

## 1. Introduction

Plants are often subjected to a variety of biotic and abiotic stresses, including drought, extreme temperatures, salinity, and other adverse environmental conditions [1]. Therefore, improving the resistance and tolerance of crops to these stresses is an important objective of breeding programs. Plants have evolved many complex molecular, cellular, physiological, and biochemical mechanisms that allow them to cope with abiotic stresses [2,3], including stomatal movement, signal perception and transduction, stress-induced gene expression, and metabolic changes [4]. When plants grow under stress conditions, transcription factors bind to specific cis-acting elements in the promoter region of target genes to regulate expression, signal transduction, and adaptation-related networks [5]. To date, diverse transcription factor families in plants, including WRKY, MYB, NAC, and bHLH, have been identified and functionally characterized regarding their involvement in biotic and abiotic stress responses [6,7,8,9].

The WRKY family of transcription factors is one of the biggest families of transcriptional regulators in plants. These plant-specific transcription factors make up an important part of the signaling network involved in the regulation of a variety of biological processes [10]. The WRKY transcription factors were named because they contain a conserved domain comprising approximately 60 amino acids as well as a conserved WRKYGQK heptapeptide sequence at the N-terminus [11]. In 1994, the first WRKY transcription factor (SPF1) was reported to mediate sucrose-regulated gene expression in sweet potatoes [12]. A subsequent study identified WRKY1, WRKY2, and WRKY3 in parsley (*Petroselinum crispum*) as additional WRKY transcription factors and revealed for the first time the importance of these types of transcription factors for regulating plant responses to pathogens [13]. The WRKY transcription factors in various plant species have been studied, including *Arabidopsis thaliana*, rice, tobacco, oat, cucumber, and moss. Moreover, genes encoding WRKY-like proteins have been detected in protozoa (*Giardia lamblia* and *Dictyostelium discoideum*) and green algae (e.g., *Chlamydomonas reinhardtii*), indicative of the ancient origin of the *WRKY* gene family [14]. Earlier studies confirmed that WRKY transcription factors contribute to plant growth and development and various biotic and abiotic stress responses. Therefore, the regulatory functions and networks of WRKY transcription factors have been a major focus of the research conducted by plant molecular biologists. In this review article, the roles of WRKY transcription factors in plant biotic and abiotic stress responses, plant nutrient stress responses, and plant secondary metabolism are described, while the regulatory mechanisms and networks associated with WRKY transcription factors are summarized.

## 2. Structural Features and Classification of the WRKY Transcription Factors

**Figure 1:**
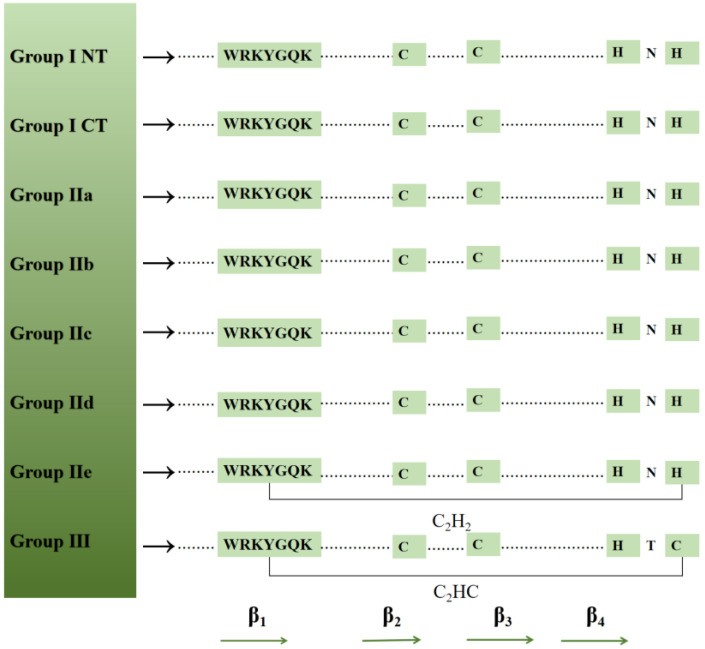
The WRKY domain. The WRKY motif as well as the cysteine and histidine residues that form zinc fingers are shown. Four β-chains are indicated by arrows. Group Ⅰ NT and CT represent the N-terminal and C-terminal WRKY domains of Group I WRKY proteins.

**Figure 2:**
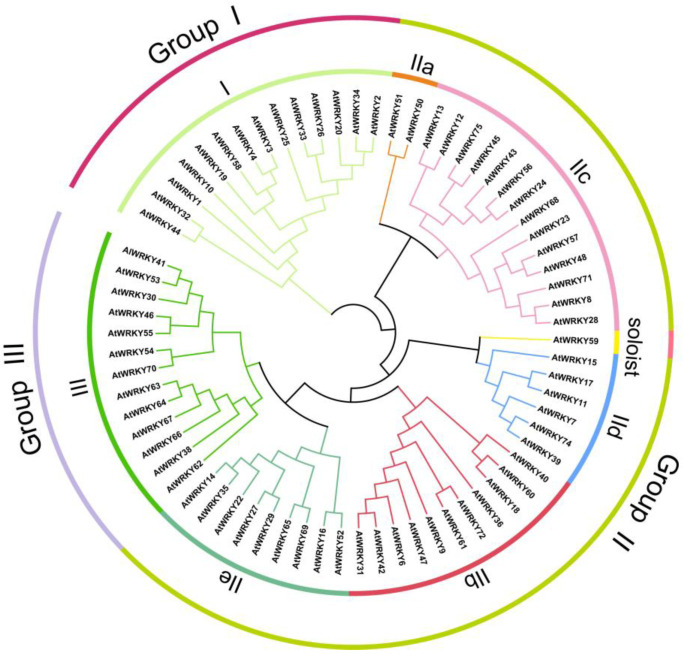
Arabidopsis WRKY gene family phylogenetic tree.

The WRKY transcription factors are key regulatory proteins that respond to biotic and abiotic stresses and regulate physiological processes and development [15]. The WRKY family, which was originally identified in sweet potatoes, consists of proteins that contain either one or two WRKY domains. The WRKY domain forms four β-sheets and consists of a conserved WRKYGQK sequence motif as well as a zinc finger (C_2_H_2_ or C_2_HC) structure [16]. These transcription factors regulate the transcription of the downstream target genes by binding specifically to the W-box [(T)TGAC(C/T)] cis-element in the gene promoter [17]. According to the number of WRKY domains and zinc-finger motifs, WRKY transcription factors have been divided into Groups I, II, and III. [17,18]. Specifically, the proteins with two WRKY domains and a C-terminal domain that plays an auxiliary role in the binding to DNA belong to Group I. The proteins in Group II and Group III contain only one WRKY domain and are distinguished by the presence of C_2_H_2_ and C_2_HC motifs, respectively [18]. Group II (i.e., the largest group of WRKY transcription factors) was further subdivided into five subfamilies (a–e) on the basis of the diversity in the conserved structural motifs other than the WRKY domain [19,20]. Group III WRKY transcription factors are associated with plant responses to a variety of diseases and insect pests and are exclusive to higher plants [20] (Figure 1). This is also confirmed by the phylogenetic analysis of the model plant *A. thaliana* (Figure 2). The WRKY proteins also contain potential basic nuclear localization signals, leucine zippers, as well as serine–threonine-rich, glutamine-rich, and proline-rich regions. The differences in the transcriptional regulatory functions of transcription factors may be due to specific structures (e.g., kinase region) [21].

## 3. Biological Functions of WRKY Transcription Factors in Plants

Recent comprehensive studies on WRKY family members have shown that these transcription factors are involved in a variety of processes related to biotic and abiotic stress responses (e.g., hormone signaling) [22] while also regulating the expression of downstream genes to promote or inhibit the synthesis of the related proteins [23]. Many WRKY transcription factor family members have been isolated from a variety of plants, including 74 in *A. thaliana*, 68 in sorghum, and more than 100 in rice, soybean, and other higher plants [10].

### 3.1. Regulatory Roles in Plant Biotic Stress Responses

Infestations by herbivorous insects and pathogen infections are among the main biotic stresses encountered by plants. During long-term evolution, plants formed two defense mechanisms that protect against biotic stresses. The first mechanism, which affects plant defenses against pathogen infections, mediates the restriction of the pathogen to the damaged site to prevent the infection from spreading (i.e., systemic acquired resistance). The second mechanism, which is induced by external stressors, leads to signaling molecules rapidly activating salicylic acid (SA) pathways. Signal transduction pathways, such as those associated with jasmonic acid (JA) and ethylene (ET), modulate the transcription of related genes and modify proteins, leading to the resistance to various biotic stresses [24,25,26]. Plants produce a suite of preformed secondary metabolites and antibacterial compounds that have inhibitory effects on potential pathogens, but they also possess the following two active defense mechanisms: pathogen-associated molecular-pattern (PAMP)-triggered immunity (PTI) and effector-triggered immunity (ETI) [27,28,29,30] (Table 1).

Shinde et al. reported that WRKY1 positively regulates the resistance of wild tomato (*Solanum arcanum*) to early blight caused by *Alternaria solani*. Another study indicated SaWRKY1 clearly up-regulates the expression of *XTH5* and *MYB2*, which influences the disease phenotype [31]. Moreover, the expression levels of 10 *WRKY* genes, including *PnWRKY9*, increase in the root. The overexpression of *PnWRKY9* in transgenic tobacco plants results in a significant increase in the resistance to *F. solani*, while the RNAi-mediated decrease in *PnWRKY9* expression increases the susceptibility of *P. notoginseng* leaves to *F. solani*. Furthermore, PnWRKY9 and the JA signaling pathway synergistically affect the disease resistance of transgenic tobacco [32]. The overexpression of the rice (*Oryza sativa*) *OsWRKY45* transcription factor gene results in increased resistance to *Magnaporthe oryzae* [33]. Twenty-five *LrWRKY* genes were identified during a previous analysis of the *Lilium regale* transcriptome, including fifteen *LrWRKY* genes whose expression is significantly induced by *Botrytis cinerea*. Compared with the wild-type control, transgenic *A. thaliana* plants overexpressing *LrWRKY39* and *LrWRKY41a* are more resistant and sensitive to *B. cinerea*, respectively [34]. However, the expression of *FvWRKY42*, which was isolated from wild strawberry (*Fragaria vesca*) (Heilongjiang No. 3; diploid), is induced by multiple stresses; the encoded transcription factor interacts with various stress response-related proteins. The overexpression of *FvWRKY42* in *A. thaliana* leads to increased powdery mildew resistance because of the associated increase in fungal cell death, sporulation, and delayed mycelial growth [35]. In *Panax notoginseng* treated with methyl jasmonate (MeJA) and infected with *Fusarium solani*, the ectopic expression of *AtWRKY18* results in up-regulated *PR* gene expression and resistance to the bacterial pathogen *Pseudomonas syringae* [36]. An earlier analysis of the interaction between AkWRKY and the W-box of *AkNBS* genes in three-leaf akebia (*Akebia trifoliata*) showed that AkWRKY24 can enhance disease resistance by positively regulating the expression of *AkNBS18* [37]. In *A. thaliana*, more than two-thirds of the identified AtWRKY members are affected by bacterial infections and SA treatments, suggestive of their key roles in biotic stress responses [38,39,40,41].

Earlier studies have shown that WRKY transcription factors can function both upstream and downstream of hormones and contribute to the antagonistic effects between SA and JA/ET, while also controlling developmental processes via auxin, cytokinin, and brassinosteroids [50]. Signaling pathways mediated by SA are associated with the resistance to biotrophic and semi-biotrophic pathogens, whereas JA/ET signaling pathways are typically associated with the resistance to necrotrophic pathogens [33]. In a study on the potential effects of ET during an infection of the *A. thaliana* EIN3/EIL1 double mutant, Rishmawi et al. observed that WRKY75 is directly regulated by ET. In the presence of ACC, *WRKY75* expression is up-regulated at 7 days post-infection. Moreover, ET has dual roles in infected rapeseed plants and contributes to root defenses against pathogens [51]. The overexpression of *EjWRKY17* in loquat (*Eriobotrya japonica*) promotes abscisic-acid (ABA)-mediated stomatal closure under drought conditions and significantly up-regulates the expression of ABA biosynthesis genes and genes related to stress responses [52]. The overexpression of the SA-inducible gene *PtrWRKY89* increases *PR* gene expression and the resistance of transgenic cottonwood trees to pathogens [53]. Most WRKY transcription factors are negative regulators, but a few are known to function as positive regulators. In peanut (*Arachis hypogaea*) plants, *AhWRKY7*, *AhWRKY8*, and *AhWRKY13* expression levels are down-regulated in response to SA and JA, which is in contrast to the up-regulated expression of the *AhWRKY1* and *AhWRKY12* genes that encode transcription factors that modulate SA and JA signal transduction pathways, reflecting their importance for disease resistance [54].

### 3.2. Regulatory Roles in Plant Abiotic Stress Responses

In addition to various biotic stresses, plants are exposed to abiotic stresses throughout their growth period, including high and low temperatures, salinity, drought, and nutrient deficiency [55]. These stresses, which can occur simultaneously, have detrimental effects on plant physiological and biochemical processes and severely restrict growth and development. The expression of *WRKY* genes is induced in response to different abiotic stresses, implying they likely influence abiotic stress tolerance. The precise regulation of WRKY proteins during plant stress responses is associated with the establishment of a complex signaling network. Given the crucial functions of WRKY proteins in plant abiotic stress responses, they are probably key contributors to stress tolerance [56,57]. Several recent investigations indicated that manipulating WRKY transcription factor levels in genetically modified plants (i.e., gene knockout or overexpression) alters specific stress responses. Some abiotic stresses strongly and rapidly induce the expression of numerous WRKY-encoding genes (e.g., drought or salinity, low and high temperatures, or osmotic stress) [10] (Table 2).

#### 3.2.1. Temperature Stress

Frequent changes in temperature and extreme weather conditions worldwide result in high- and low-temperature stresses. Thus, temperature stress is one of the main abiotic stresses affecting plants. Temperature extremes alter plant physiological indices and affect plant growth and development, ultimately resulting in economic losses for agriculturally important plant species. Therefore, plant cells must be protected from the detrimental effects of temperature changes. Accordingly, temperature-stress-induced changes in plants should be elucidated to provide the theoretical basis for crop breeding and the development of improved agronomic practices to increase agricultural production [58,59]. Many WRKY transcription factors participate in plant responses to temperature stress by regulating the expression of related genes. For example, the expression of 10 *OsWRKY* genes in rice is significantly altered by different environmental stresses, including cold/heat, salinity (sodium chloride treatment), and simulated drought (e.g., polyethylene glycol treatment) [60]. Rizhsky et al. determined that most tobacco *WRKY* gene expression levels are altered by cold/heat stresses [61].

High-temperature stress can damage plant cell membranes and disrupt reactive oxygen species (ROS) homeostasis, resulting in changes to plant structures and functions [62]. Recent research showed that in medicinal dandelion, *WRKY* gene expression is up-regulated substantially more under high-temperature stress conditions than in response to low-temperature stress [63]. Dang [64] reported that the expression of *CaWRKY40* increases significantly in the leaves of pepper (*Capsicum annuum*) plants grown at high temperatures. In *A. thaliana*, the expression levels of both *AtWRKY25* and *AtWRKY26* increase following a high-temperature treatment, whereas the expression of *AtWRKY33* decreases; however, mutations to all three genes increase the sensitivity of *A. thaliana* plants to high-temperature stress [65]. The initial stage of the wheat streak rust infection of wheat is characterized by a significant increase in the *TaWRKY70* expression level at elevated temperatures. This finding reflects the positive correlation between *TaWRKY70* expression and the resistance of wheat seedlings to heat stress as well as the likely activation of the SA and ET signaling pathways during the initial stage of the infection [66]. Wu et al. isolated a heat-induced differentially expressed lily (*Lilium longiflorum*) gene belonging to WRKY Group IIe (*LlWRKY22*). This gene encodes a protein involved in the mechanism underlying heat tolerance. Additional research revealed that the overexpression of *LlWRKY22* increases the heat tolerance of transgenic plants and up-regulates the expression of *AtDREB2A*, *AtDREB2B*, *AtDREB2C*, and *AtJUB1*. However, the LlWRKY22 regulatory pathway is also associated with the ABA signaling pathway [67].

The analysis of the cotton (*Gossypium hirsutum*) transcriptome identified 10 *WRKY* genes with up-regulated expression levels under low-temperature stress conditions [68]. Using RNA-seq and chromatin immunoprecipitation sequencing data, Guo et al. showed that SlWRKY33 directly targets and induces the expression of multiple genes encoding kinases, transcription factors, and molecular chaperones, including *CDPK11*, *MYBS3*, and *BAG6*, thereby enhancing cold tolerance [69]. The overexpression of *OsWRKY76* in rice (*O. sativa*) positively affects cold tolerance [70]. Furthermore, AtWRKY34 negatively regulates the sensitivity of mature pollen grains to cold stress by inhibiting the expression of CBF pathway genes [71].

#### 3.2.2. Drought Stress and Salt Stress

Two major environmental factors that significantly limit global crop production are drought and high salinity. More specifically, these two environmental stresses lead to an intracellular ionic and osmotic imbalance while also interfering with photosynthetic activities, cellular energy consumption, and the redox balance. The WRKY family of transcription factors is involved in the regulatory system that links the perception and transduction of environmental signals with adaptive cellular responses. Many WRKY transcription factors were identified as the primary factors regulating molecular programming, which can enhance plant stress resistance. Some WRKY transcription factors are related to the drought and salt stress tolerance of *A. thaliana*, rice, and other plants. Several established mechanisms that mediate drought and salinity tolerance are affected by ABA signaling. More specifically, ABA has been implicated in plant responses to drought conditions, and components of the ABA signaling pathway in the nucleus are targeted by signals from plastids and mitochondria, which affects the retrograde signal transduction in plant cells [72,73].

Yan et al. detected induced *GhWRKY17* expression in plants treated with various stresses (i.e., drought, salt, H_2_O_2_, and ABA). On the basis of the germination rate, root growth, survival rate, leaf water loss, and chlorophyll content, constitutive *GhWRKY17* expression significantly decreases the tolerance of *Nicotiana benthamiana* to drought and salt stresses [74]. However, *IgWRKY50* and *IgWRKY32* in *Iris germanica* encode positive regulators that enhance the drought resistance of *A. thaliana* transgenic plants by modulating the ABA signal transduction pathway [75]. The expression of *GsWRKY20* in transgenic *A. thaliana* plants leads to increased drought tolerance [76]. In a separate study, *GmWRKY6* overexpression was confirmed to improve the resistance of *Lotus japonicus* to salt stress, whereas the overexpression of *GmWRKY12* enhances the drought resistance and salt tolerance of hairy roots [77]. Many cotton *WRKY* genes play a crucial regulatory role during the stress responses of model plants. For example, the overexpression of *GhWRKY34* and *GhWRKY41* reportedly increases the salt tolerance of *A. thaliana* and the drought tolerance of tobacco [78,79]. Additionally, GhWRKY1 is a transcriptional regulator related to the drought tolerance of upland cotton (*G. hirsutum*). The overexpression of genes encoding GhWRKY1-like proteins in *A. thaliana* increases drought tolerance by manipulating the synthesis of ABA and interactions with several cis-elements [80].

Under drought conditions, *PmWRKY6*, *PmWRKY10*, and *PmWRKY30* expression levels are up-regulated in different plants and tissues, while *PmWRKY22* expression is down-regulated. Compared with wild-type tobacco, *PmWRKY31*-expressing transgenic tobacco plants contain less malondialdehyde but more proline. Significant increases in the expression of the related genes have been detected in *PmWRKY31*-expressing transgenic tobacco, which leads to an increase in drought tolerance [81]. Moreover, AtWRKY33 is an upstream regulator of AtCYP94B1 in *A. thaliana*; mutations to *AtWRKY33* result in decreased suberin levels and salt-sensitive phenotypes, further confirming that AtWRKY33-mediated AtCYP94B1 regulation is part of the salt tolerance mechanism [82]. The *A. thaliana* genes *AtWRKY21*, *AtWRKY46*, *AtWRKY54*, and *AtWRKY70* encode regulators of osmotic stress responses [83,84]. The DgWRKY1 transcription factor positively regulates the salt stress response [85]. A previous study indicated that salt stress induces *PeWRKY1* expression in *Populus euphratica* and that the positive effect of *PeWRKY1* expression on the salt tolerance of transgenic tobacco lines is associated with the promotion of Na^+^ efflux from the root cells [86].

#### 3.2.3. Nutrient Stress

Normal plant growth and development depends on the uptake of various nutrients, including nitrogen, phosphorus, and potassium. Plant morphogenesis is severely affected by the lack of essential elements. Phosphorus is one of the essential nutrients required by plants, representing approximately 0.2% of the plant dry weight. In plants and animals, phosphorus helps regulate key metabolic pathways as well as many enzymatic reactions. Plants absorb phosphorus in the form of phosphate. The first WRKY transcription factor revealed to be associated with phosphorus deficiency was *AtWRKY75*. Under phosphorus-deficient conditions, *AtWRKY75* expression is strongly induced in *A. thaliana*. The inhibited expression of this gene increases the sensitivity of plants to phosphorus stress. Furthermore, the uptake of phosphorus by plants decreases in low-phosphorus environments [87]. Among the *WRKY* genes in cotton (*Gossypium barbadense*), *GbWRKY1* regulates plant responses to phosphorus deficiency. The overexpression of *GbWRKY1* in *A. thaliana* can decrease the severity of the effects of phosphorus deficiency, induce the accumulation of phosphorus, and promote lateral root development and phosphatase activity [88]. Shen et al. identified WRKY33 as a negative regulator of phosphorus deficiency-induced root architecture remodeling in *A. thaliana*. More specifically, it controls the transcription of *ALMT1* under phosphorus-deficient conditions, thereby modulating the accumulation of Fe^3+^ in the root tips and inhibiting root growth [89].

Nitrogen and potassium are also critical nutrients required by plants. In rice (*O. sativa*), the expression levels of many *WRKY* genes are modified (up-regulated or down-regulated) by low-nitrogen stress [90]. Of the wheat (*Triticum aestivum*) *WRKY* genes, 10 (e.g., *WRKY4*, *WRKY6*, *WRKY12*, and *WRKY18*) encode positive regulators of plant responses to potassium deficiency, whereas the other genes encode negative regulators [91]. The WRKY transcription factors are widely implicated in alleviating the deleterious effects of plant nutrient stress, especially nitrogen, phosphorus, and potassium deficiencies. Furthermore, specific micronutrients are important for plant growth and developmental processes. Earlier research showed WRKY transcription factors are involved in altering plant activities in response to a lack of trace elements. For example, under iron stress conditions, WRKY46 can directly regulate the transcription of *VITL1* (*vacuolar iron transporter1-like1*) by specifically binding to the W-box region in the gene promoter, which affects iron uptake and transport in plants [92]. In response to boron stress, the WRKY transcription factors in *A. thaliana* induce the expression of *NIP5;1* (*nodulin 26-like intrinsic protein 5;1*), allowing plants to adapt to the limited availability of boron, indicating that WRKY6 can regulate root tip gene expression under boron-deficient conditions [93]. Many WRKY family members that regulate plant responses to micronutrient stress have been identified. In wheat (*T. aestivum*), an exposure to calcium stress up-regulates the expression of four *WRKY* genes, including *WRKY18*, but has the opposite effect on *WRKY6* expression. Under zinc stress conditions, the expression of six *WRKY* genes (e.g., *WRKY3*) is up-regulated in wheat, but the expression of three other *WRKY* genes (e.g., *WRKY17*) is down-regulated [91].

The WRKY transcription factors also participate in the regulation of plant responses to other elements, some of which have beneficial effects. In *A. thaliana*, AtWRKY46 is a negative regulator associated with plant responses to aluminum stress, which inhibits *AtWRKY46* expression. If *A. thaliana* plants are treated with toxic levels of aluminum, WRKY46 binds directly to the W-box of the *ALMT1* (*aluminum-activated malate transporter 1*) promoter, thereby negatively regulating *ALMT1* expression and improving the resistance to aluminum stress [94]. The expression of *PyWRKY75*, which was isolated and cloned from poplar (*Populus yunnanensis*), significantly promotes cadmium uptake and accumulation in trees, with cadmium levels that are 51.32% higher in *PyWRKY75*-overexpressing trees than in the wild-type controls. Antioxidants, such as peroxidase (POD), superoxide dismutase (SOD), catalase (CAT), ascorbate peroxidase (APX), ascorbic acid (AsA), reduced glutathione (GSH), and phytochelatins (PCs), can increase the cadmium stress tolerance of transgenic poplar trees and accumulate differentially in the roots, shoots, and leaves [95]. These findings provide important insights into the contributions of WRKY transcription factors to plant responses to nutrient stress.

**Table 2:** Regulation of WRKY transcription factors in abiotic stress responses.

Type of Stress	Species	Gene	References
High temperature	Pepper (*Capsicum annuum*)	*CaWRKY40*	[64]
	Arabidopsis (*Arabidopsis thaliana*)	*AtWRKY25, AtWRKY26*, *AtWRKY33*	[65]
	Wheat (*Triticum aestivum*)	*TaWRKY70*	[66]
	Lily (*Lilium browniivar*)	*LlWRKY22*	[67]
Low temperature	Cultivated tomato (*Solanum lycopersicum*)	*SlWRKY33*	[69]
	Rice (*Oryza sativa*)	*OsWRKY76*	[70]
	Arabidopsis (*Arabidopsis thaliana*)	*AtWRKY34*	[71]
Drought	German iris (*Iris germanica*)	*IgWRKY50, IgWRKY32*	[75]
	Wild soybean (*Glycine soja*)	*GsWRKY20*	[76]
	Soybean (*Glycine max*)	*GmWRKY12*	[77]
	Upland cotton (*Gossypium hirsutum*)	*GhWRKY1*	[80]
	Pinus massoniana (*Pinus massoniana*)	*PmWRKY31*	[81]
Salt	Soybean (*Glycine max*)	*GmWRKY6*	[77]
	Cotton (*Gossypium hirsutum*)	*GhWRKY34, GhWRKY41*	[78,79]
	Arabidopsis (*Arabidopsis thaliana*)	*AtWRKY33*	[82]
	Chrysanthemum (*Dendranthema grandiflorum*)	*DgWRKY1*	[85]
	Diversiform-leaved poplar (*Populus euphratica*)	*PeWRKY1*	[86]
Phosphorus	Arabidopsis (*Arabidopsis thaliana*)	*AtWRKY75*	[87]
	Gossypium barbadense (*Gossypium barbadense*)	*GbWRKY1*	[88]
Aluminum	Arabidopsis (*Arabidopsis thaliana*)	*AtWRKY46*	[93]
	Soybean (*Glycine max*)	*GmWRKY21*	[94]
Cadmium	Poplar (*Populus yunnanensis*)	*PyWRKY75*	[95]
Iron	Rice (*Oryza sativa*)	*OsWRKY74*	[96]

#### 3.2.4. Oxidative Stress

Of the other stresses that affect plants, oxidative stress is one of the most harmful [97]. In plants, the four major types of ROS are singlet oxygen (O_2_), hydroxyl radicals (OH·), superoxide anions (O_2_^−^), and hydrogen peroxide (H_2_O_2_). Therefore, the active oxygen signaling network modulated by WRKY transcription factors has important biological functions in plants (Table 3).

Various WRKY transcription factors in *A. thaliana* are activated by H_2_O_2_ stress. Cytoplasmic ascorbate peroxidase 1 (Apx1) is an H_2_O_2_ scavenger. An earlier microarray analysis of Apx1-deficient *A. thaliana* plants by Rizhsky et al. revealed that the production of two zinc-finger proteins (Zat12 and Zat7) and WRKY25 increases in *Apx1*-knockout plants cultured under controlled conditions. Thus, Zat12 plays an important role in the signal transduction network mediating the response of *A. thaliana* to oxidative stress. Specifically, Zat12 is required for the expression of *Zat7*, *WRKY25*, and *Apx1* under oxidative stress conditions [97].

Studies have shown that the adaptation of *A. thaliana* to underwater hypoxic conditions involves the interaction between WRKY33 and WRKY12, which positively regulates the expression of the ethylene-responsive factor VII gene *RAP2.2*; the overexpression of *WRKY33* and *WRKY12* increases plant resistance to hypoxia [98]. Another study demonstrated that WRKY53 can interact directly with a mitogen-activated protein kinase (MEKK1) and regulate the expression of *CAT1*, *CAT2*, and *CAT3*, which help mediate antioxidant defenses [99]. The activated expression of *AtWRKY53* inhibits stomatal closure by decreasing the H_2_O_2_ content and induces stomatal opening by promoting starch degradation [100]. The overexpression of *GhWRKY68* impairs the ability of plants to tolerate oxidative stress under both drought and saline conditions [101]. An earlier study by Yan et al. compared ROS levels in *GhWRKY68*-overexpressing transgenic and wild-type plants under normal and stressed conditions. Under normal conditions, both H_2_O_2_ and O_2_^−^ accumulated at low levels, and there were no significant differences between the wild-type and transgenic lines; however, after the exposure to drought and saline conditions, the transgenic lines accumulated more H_2_O_2_ and O_2_^−^, implying that GhWRKY17 increased ROS levels after the exposure to drought and salt stresses [74]. Cold and excessive salinity can substantially induce *VvWRKY28* expression in grapes (*Vitis vinifera*). The expression of *VvWRKY28* can considerably increase the tolerance of *A. thaliana* to low temperatures and high salinity, likely because of the associated increase in SOD, POD, and CAT activities [102]. Therefore, WRKY transcription factors have important biological functions influencing the active oxygen signaling network in plants.

## 4. Resistance-Related Regulatory Effects of WRKY Transcription Factors on Plant 

### Secondary Metabolism

The role of WRKY transcription factors during the biosynthesis of natural products [103] has recently become a topic of interest among researchers. The WRKY family of transcription factors is involved in plant metabolic processes that result in the production of many secondary metabolites, including lignin, plant hormones, phenols, alkaloids, terpenes, and flavonoids. Secondary metabolites have crucial functions affecting plant growth, development, and responses to environmental stimuli (Table 4).

In *Isatis indigotica*, *IiWRKY34* expression levels increase following drought and salt treatments, with IiWRKY34 subsequently binding to the W-box in the promoter region of *Ii4CL3*, which encodes the key rate-limiting enzyme for lignin biosynthesis. The accumulation of lignin increases the biological activity and stress resistance of plants [104]. Maize (*Zea mays*) plants accumulate terpenoid phytoalexins, liquiritigenin, and zeatin in response to various stimuli. The biosynthesis of maize terpenoid phytoalexins is regulated by ZmWRKY79, which is a potential major regulator of stress responses through its effects on plant hormone metabolism or signal transduction and ROS scavenging [105].

Research on the regulatory effects of WRKY transcription factors on alkaloid synthesis has mostly focused on the indole alkaloid biosynthesis pathway. The CjWRKY1 transcription factor in *Coptis japonica* was the first transcription factor confirmed to regulate alkaloid production. More specifically, it affects the biosynthesis of benzylisoquinoline alkaloids [106]. Moreover, CrWRKY1 controls the biosynthesis of terpene indole alkaloids, which have anti-tumor effects, by regulating the expression of the tryptophan decarboxylase (TDC)-encoding gene in *Catharanthus roseus* [107]. In *Ophiorrhiza pumila*, OpWRKY6 negatively regulates the biosynthesis of camptothecin in the iridoid pathway and the shikimate pathway by directly down-regulating the expression of *OpGES*, *Op10HGO*, *Op7DLH*, and *OpTDC* [108].

An earlier analysis of terpenoid biosynthesis in tomato (*Solanum lycopersicum*) revealed that SlWRKY71 regulates the expression of the terpenoid synthase gene following a JA treatment [109]. Jasmonide is a diterpenoid plant antitoxin with allelopathic effects and is widely distributed in feathery bryophytes. Examinations of cis-acting elements and binding sites indicated that stress induces *CpWRKY* expression, which influences jasmonide biosynthesis [104]. Artemisinin is currently the most effective substance for treating malaria. The *AaWRKY1* gene, which was isolated from secretory glandular trichomes, encodes a protein that binds to the W-box of the *ADS* promoter to activate transcription and promote artemisinin biosynthesis [110]. The cDNA sequences of many *WRKY* genes in *Gossypium arboreum* have been isolated. One of these genes, *GaWRKY1*, encodes a protein containing a single WRKY domain and a putative leucine zipper at the N-terminus. In suspension cells, fungal elicitors and MeJA induce *GaWRKY1* and *CAD1-A* expression and sesquiterpene aldehyde biosynthesis [111].

In addition, some WRKY transcription factors can negatively regulate flavonoid synthesis in plants. For example, the heterologous expression of the *Brassica napus* gene *BnWRKY41-1* in *A. thaliana* rosette leaves reportedly leads to decreases in the anthocyanin content [112]. Long-term studies on rice (*O. sativa*) have shown that OsWRKY13 up-regulates *CHS* expression and controls flavonoid phytoalexin biosynthesis. Furthermore, WRKY proteins may be important for increasing the expression of *OsWRKY13* and genes involved in defense responses to pathogens [113]. Recent research revealed a new disease defense mechanism, the WRKY–MAPK pathway, which protects plants against pathogens by promoting flavonoid biosynthesis [114].

## 5. WRKY Transcription Factor Regulatory Network

**Figure 3:**
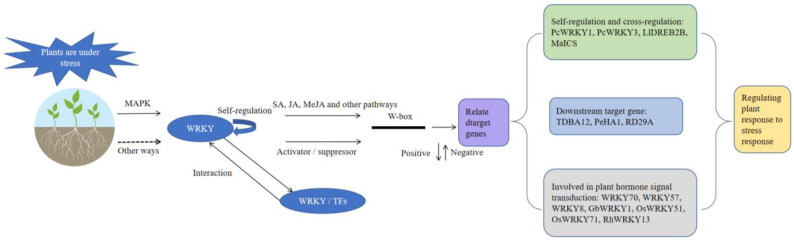
Mechanism underlying the regulatory effects of WRKY transcription factors on plant stress responses.

At the DNA level, each WRKY transcription factor recognizes and binds to the W-box domain in its target genes, which may include its own gene, to activate or inhibit transcription. Interactions between WRKY transcription factors and the downstream target genes give rise to a complex WRKY regulatory network. At the protein level, WRKY transcription factors and diverse regulatory proteins coordinately respond to a variety of environmental stresses (Figure 3).

### 5.1. Self-Regulation and Cross-Regulation by WRKY Transcription Factors

The promoters of WRKY transcription factor target genes and the promoters of most WRKY transcription factor genes contain W-box elements. The WRKY transcription factors control various stress responses via self-regulation as well as the cross-regulation of other WRKY transcription factors [114,115]. For example, the promoter region of *PcWRKY1* has multiple W-box elements that mediate the binding of WRKY transcription factors [116]. In addition to binding to the W-box in its own promoter, PcWRKY1 can also bind to the W-box in the *PcWRKY3* promoter region [117]. Wu et al. recently identified LlWRKY22 as a novel regulator of lily plant responses to high-temperature stress. Specifically, it induces the expression of its own gene to form a positive feedback loop, while also activating the expression of *LlDREB2B*, which encodes a core regulator of heat stress responses [67]. In *P. euphratica*, PeWRKY1 binds to the W-box of the *PeHA1* and *PeMAX2* promoters to regulate expression. Therefore, PeWRKY1 improves ion homeostasis in *P. euphratica* in two ways [76]. In banana (*Musa acuminata*), MaWRKY21 binds directly to the W-box of the *MaICS* promoter and negatively regulates transcription, thereby decreasing enzyme activity levels [118].

### 5.2. WRKY Transcription Factors Interact with the Downstream Target Genes

The WRKY transcription factors bind specifically to the W-box (TTGACC/T) of the downstream target genes via their WRKY domain [10,119,120]. For example, under disease stress conditions, WRKY transcription factors bind to the W-box of the tobacco chitinase gene *TDBA12* [121]. In *A. thaliana*, AtWRKY8 mediates the resistance to *Phytophthora infestans* by interacting with the genes downstream of the MAPKKKα–MEK2–WIPK signaling cascade, which results in H_2_O_2_ accumulation and the apoptosis of plant cells [122]. In *P. euphratica*, PeWRKY1 binds to the W-box element in the promoter of *PeHA1* (encoding a plasma membrane H^+^-ATPase) to increase transcription. The PeWRKY1–PeHA1/PeMAX2–PeGRP2 signaling network that maintains the ionic balance in *P. euphratica* provides important insights into the molecular basis of the *P. euphratica* response to salinity stress [87]. Furthermore, AtWRKY6, AtWRKY42, and AtWRKY72 control plant responses to nutrient stress (e.g., low phosphorus levels) by regulating the expression of the downstream target genes [123,124]. The W-box is a cis-acting element that is generally associated with up-regulated expression, but the binding of a WRKY transcription factor to the W-box in the *AtWRKY18* promoter in *A. thaliana* results in down-regulated expression [47]. The WRKY8 transcription factor regulates the expression of the downstream genes, such as *RD29A*, after interacting with the VQ9 protein [125].

### 5.3. WRKY Transcription Factors Involved in Plant Hormone Signal Transduction

The WRKY transcription factors are critical components of the plant signaling network that regulates growth and development under biotic and abiotic stress conditions. These transcription factors may function as activators or repressors in a transcription factor network modulating the transduction of signals from organelles and the cytoplasm to the nucleus. They may have roles upstream and downstream of hormones; contribute to the antagonism among the SA, JA, and ET pathways; and control development-related processes through auxin, cytokinin, and brassinosteroids. The SA-mediated signaling pathway is generally associated with the resistance to biotrophic and semi-biotrophic pathogens, whereas JA/ET signaling pathways are associated with the resistance to necrotrophic pathogens [32,33,34]. For example, WRKY70 activates the expression of SA-responsive genes but has the opposite effect on JA-responsive genes. Thus, it integrates the signals from these antagonistic pathways [126]. He et al. [127] determined that the synergistic effects of OsWRKY51 and OsWRKY71 inhibit gibberellin signaling in rice (*O. sativa*) seed aleurone cells.

Cytokinins and ABA are key hormones influencing plant growth, development, and stress responses. In rose (*Rosa hybrida*), RhWRKY13 binds to the promoters of the *cytokinin oxidase/dehydrogenase 3* (*RhCKX3*) gene and the ABA-responsive *ABA INSENSITIVE4* (*RhABI4*) gene, leading to the simultaneous inhibition of gene expression in rose petals [128]. Increased cytokinin contents and inhibited ABA responses contribute to the protection of plants from *B. cinerea* infections. The WRKY2, WRKY18, WRKY40, WRKY60, and WRKY63 transcription factors reportedly modulate the expression of genes encoding ABA response element-binding factors (ABFs/AREBs) by binding to the W-box sequence of the corresponding gene promoters. The ABA signal in the nucleus is a target of plastid and mitochondrial signals which influence retrograde signaling in plant cells [39,129,130]. Studies have shown that WRKY15 is involved in the transduction of signals from the mitochondria to the nucleus and that auxin and cytokinin control plant development with the assistance of WRKY transcription factors [131]. The *A. thaliana* WRKY57 transcription factor can interact with the JA signaling pathway inhibitors JAZ4/JAZ8 and the auxin signaling pathway inhibitor IAA29, which links the JA- and auxin-mediated plant leaf senescence signaling pathways. Furthermore, WRKY57 binds directly to the promoters of the ABA-related genes *RD29A* and *NCED3* to regulate plant drought resistance [129]. In *A. thaliana*, WRKY8 aids in the regulation of antiviral responses by regulating ABA and ET signal transduction pathways [125]. Previous research indicated that GbWRKY1 influences cotton resistance to Verticillium wilt via the SA, JA, and ET signaling pathways. Subsequent investigations suggested that GbWRKY1 mainly regulates plant growth and development by participating in ET and other signaling pathways and affecting cytoprotective enzyme activities and resistance-protein-coding gene transcription to improve plant resistance to Verticillium wilt [88].

### 5.4. Other Regulatory Networks

In addition to the W-box element, WRKY transcription factors can also bind to other sequences. For example, the aging-related transcription factor AtWRKY53 must interact with MEKK1, a MAP kinase, before it can bind to the W-box element [132]. In barley (*Hordeum vulgare*), WRKY transcription factors related to glucose metabolism can bind to SURE (sugar responsive) elements to facilitate starch synthesis [133]. Zhang et al. identified 104 WRKY33 target genes in *A. thaliana* under waterlogging stress conditions. A novel cis-element (TCTCTC) designated as the TC box is the major motif in the target gene promoters. This cis-element differs from the W-box previously identified to interact with WRKY33. Genes with a promoter containing this element may be regulated by WRKY33 under flooding conditions. This suggests that the WRKY33 function during the plant response to flooding conditions may depend on the TC box in the promoters of the downstream target genes [134]. The PnWRKY9 recombinant protein overexpressed in tobacco (*Nicotiana tabacum*) was observed to bind specifically to the W-box sequence in the promoter of a JA-responsive and fungal-resistance-related defensin gene (*PnDEFL1*) in eggplant. According to yeast one-hybrid assays, PnWRKY9 can activate *PnDEFL1* transcription; a tobacco co-expression experiment involving β-glucosidase as a reporter verified these findings [32].

## 6. Summary and Future Outlook

Much of the research on WRKY transcription factors conducted to date has involved model plants, including arabidopsis, tobacco, rice, soybean, and other economic crops. However, the role of many WRKY transcription factors during evolution and their functional differences must be more thoroughly characterized to further improve plant stress resistance and optimize the breeding of new varieties. Although their biological functions have been extensively studied, there are still some gene functions and modes of action that are unclear. Whole-genome and transcriptome analyses will help to elucidate the functions of WRKY transcription factors during plant stress responses. Molecular biology-based techniques should be applied to mine genomic data, functionally annotate WRKY transcription factors, and clarify the synergistic response mechanisms of other transcription factors.

WRKY transcription factors in the calcium ion (Ca^2+^) signal transduction pathway associated with plant–pathogen interactions induce the expression of defense-related genes, implying it may be important for plant disease resistance [135,136]. However, the plant response and gene regulatory system mediated by the calcium ion (Ca^2+^) signal transduction pathway associated with WRKY transcription factors and plant-pathogen interactions are not well understood. Future studies should aim to integrate sequencing data with information regarding protein–DNA and protein–protein interactions to more precisely characterize the regulation of important biological functions by WRKY tran-scription factors, which may lead to the construction of a comprehensive regulatory network. A thorough understanding of the mechanisms underlying WRKY transcription factor functions at the molecular level combined with molecular marker-assisted breeding and biotechnological tools may enable researchers and breeders to develop and select enhanced varieties of economically important crops.

## Figures and Tables

**Table 1:** Regulation of WRKY transcription factors in biotic stress responses.

Plant Species	Gene	Species of Pathogenic Bacteria/Fungi	Way of Participation	References
Wild tomato (*Solanum arcanum Peralta*)	*SaWRKY1*	*Alternaria solani*	Interaction pathway between plant pathogens: ETI	[31]
*Panax notoginseng*	*PnWRKY9*	*Fusarium solani*	Methyl jasmonate (MeJA) signal transduction pathway	[32]
Rice (*Oryza sativa*)	*OsWRKY45*	*Magnaporthe oryzae*	Interaction pathway between plant pathogens: ETI	[33]
Lilium regale (*Lilium browniivar*)	*LrWRKY39*	*Botrytis cinerea*	Interaction pathway between plant pathogens: ETI	[34]
	*LrWRKY41a*		Interaction pathway between plant pathogens: ETI	
Diploid woodland Strawberry (*Fragaria vesca*)	*FvWRKY42*	*Sphaerotheca aphanis*	Interaction pathway between plant pathogens: ETI	[35]
Arabidopsis (*Arabidopsis thaliana*)	*AtWRKY18*	*Pseudomonas syringae*	Interaction pathway between plant pathogens: ETI	[36]
Three-leaf akebia (*Akebia trifoliata*)	*AkWRKY24*		Interaction pathway between plant pathogens: ETI	[37]
Arabidopsis (*Arabidopsis thaliana*)	*AtWRKY*		Interaction pathway between plant pathogens: ETI; salicylic acid (SA) signal transduction pathway	[38,39,40,41]
Cotton (*Gossypium hirsutum*)	*GhWRKY44*	*Ralstonia solanacerum*	Interaction pathway between plant pathogens: PTI, ETI; salicylic acid (SA) signal transduction pathway	[42]
Cotton (*Gossypium hirsutum*)	*GhWRKY44*	*Rhizoctonia solani*	Interaction pathway between plant pathogens: PTI, ETI; salicylic acid (SA) signal transduction pathway	[42]
Oilseed rape (*Brassica napus*)	*BnWRKY15, BnWRKY33*	*Sclerotinia sclerotiorum*	Interaction pathway between plant pathogens: ETI; salicylic acid (SA) and jasmonic acid (JA) signal transduction pathways	[43]
Wheat (*Triticum aestivum*)	*TaWRKY49, TaWRKY62*	*Puccinia striiformis*	Interaction pathway between plant pathogens: PTI; salicylic acid (SA), jasmonic acid (JA), ethylene (ET) signal transduction pathway	[44]
Tomato (*Solanum pimpinellifolium*)	*SpWRKY3*	*Phytophthora infestans*	Interaction pathway between plant pathogens: ETI	[45]
Rice (*Oryza sativa*)	*OsWRKY67*	*Bacteria blight*	Interaction pathway between plant pathogens: ETI; salicylic acid (SA) signal transduction pathway	[46]
Cucumber (*Cumis sativus*)	*CsWRKY50*	*Pseudoperonospora ubensis*	Interaction pathway between plant pathogens: PTI, ETI; salicylic acid (SA) and jasmonic acid (JA) signal transduction pathways	[47]
Banana (*Musa acuminata*)	*MaNAC5/MaWRKY1/MaWRKY2*	*Colletotrchum musae*	Interaction pathway between plant pathogens: ETI	[48]
Rice (*Oryza sativa*)	*OsWRKY53*	*Chilo suppressalis*	Interaction pathway between plant pathogens: PTI; ethylene (ET) signal transduction pathway	[49]
Arabidopsis (*Arabidopsis thaliana*)	*WRKY75*		Interaction pathway between plant pathogens: PTI; ethylene (ET) signal transduction pathway	[50,51]
Loquat (*Eriobotrya japonica*)	*EjWRKY17*		Interaction pathway between plant pathogens: ETI; abscisic-acid (ABA) signal transduction pathway	[52]
Poplar (*Populus trichocarpa*)	*PtrWRY89*		Interaction pathway between plant pathogens: ETI; salicylic acid (SA) signal transduction pathway	[53]
Peanut (*Arachis hypogaea)*	*AhWRY1*		Salicylic acid (SA), and jasmonic acid (JA) signal transduction pathway	[54]
	*AhWRY7*			
	*AhWRY8*			
	*AhWRY12*			
	*AhWRY13*			

**Table 3:** WRKY transcription factors that induce oxidative stress responses during an exposure to other stresses.

Type of Stress	Species	Gene	Signal Transduction Pathway	References
Apx1	Arabidopsis (*Arabidopsis thaliana*)	*WRKY25*	Zinc-finger protein Zat12	[97]
Flooding	Arabidopsis (*Arabidopsis thaliana*)	*WRKY33, WRKY12*	Ethylene response factor VII gene RAP2.2	[98]
	Arabidopsis (*Arabidopsis thaliana*)	*WRKY53*	MEKK1	[99]
			starch degradation	[100]
		*AtWRKY8*	MAPKKα-MEK2-WIPK signaling cascade downstream gene interaction	[101]
Drought and salt	Cotton (*Gossypium hirsutum*)	*GhWRKY68*	Accumulate more H_2_O_2_ and O_2_-	[101]
		*GhWRKY17*	Increased reactive oxygen species (ROS) levels	[74]
Low temperature and salt	Grape (*Vitis vinifera*)	*VvWRKY28*	Superoxide dismutase (SOD), peroxidase (POD), and catalase (CAT) synthesis pathways	[102]

**Table 4:** Regulatory effects of WRKY transcription factors on plant secondary metabolism.

Species	Gene	Secondary Metabolites	Regulation Mode	References
Isatis indigotica (*Isatis tinctoria*)	*IiWRKY34*	Lignin	Positive regulation	[104]
Maize (*Zea mays*)	*ZmWRKY79*	Phytohormone	Positive regulation	[105]
*Coptis japonica*	*CjWRKY1*	Alkaloid	Positive regulation	[106]
*Catharanthus roseus*	*CrWRKY1*	Terpenoid indole alkaloids	Positive regulation	[107]
Dwarf lilyturf root (*Ophiorhiz pumila*)	*OpWRKY6*	Camptothecin	Negative regulation	[108]
Tomato (*Solanum lycopersicum*)	*SlWRKY71*	Terpenoids	Positive regulation	[109]
*Artemisia annua*	*AaWRKY1*	Artemisinin	Positive regulation	[110]
Cotton (*Gossypium hirsutum*)	*GaWRKY1*	Sesquiterpene aldehyde	Positive regulation	[111]
Oilseed rape (*Brassica napus*)	*BnWRKY41-1*	Cyanidin	Negative regulation	[112]
Rice (*Oryza sativa*)	*OsWRKY13*	Flavonoids	Positive regulation	[113]

## Data Availability

Not applicable.
